# Society Meetings

**Published:** 1893-09

**Authors:** 


### Society Meetings.

### PROGRAMME OF THE SECTION ON GYNECOLOGY AND ABDOMINAL SURGERY, PAN-AMERICAN MEDICAL CONGRESS.

To be held at the Law Department of the National University, Thirteenth street, between H and I streets, N. W., Washington, D. C., September 5, 6, 7, and 8, 1893.

### EXECUTIVE OFFICERS.

Dr. W. W. Potter, Buffalo, N. Y., Executive President; Dr. Brooks H. Wells, New York, Dr. Ernest W. Cushing, Boston, Secretaries.

### OPENING SESSION, SEPTEMBER 5TH, 3 P. M.

Address of welcome, by the Executive President; The Value of Certain Methods of Surgical Treatment for Chronic Procidentia of the Uterus, Aug. P. Clarke, Cambridge, Mass.; The Coördination of the Muscles Closing the Urethra, Vagina, and Rectum, and its Application to the Precise Diagnosis and Surgical Treatment of Injuries of the Pelvic Floor, A. W. Abbott, Minneapolis, Minn.; The Intra-uterine Tampon, Andrew F. Currier, New York; Gynecological Treatment in Sterile Women, De Saussure Ford, Augusta, Ga.; Shortening of the Round Ligaments in Retroposition of the Uterus, T. Johnson Alloway, Montreal, Can.; The Relation of Urinary Conditions to Gynecological Surgery, Chas. P. Noble, Philadelphia, Pa.; Ectopic Pregnancy, Joseph Hoffmann, Philadelphia, Pa.; Two Cases of Ectopic Gestation with Unusual Complications—Laparatomy—Recovery—T. H. Hawkins, Denver, Col.; The Treatment of Extra-uterine Pregnancy after the Viability of the Child, with Report of Two Cases, Joseph Taber Johnson, Washington, D. C.; Observacions sobre un Caso de Preñez Extrauterina (Tubaria derecha) Operado en el Hospital de San Salvador por el Dr. José Antonio Delgado, J. Antonio Delgado, Guatemala City, Guatemala.

### MORNING SESSION, SEPTEMBER 6TH.

The Technique of Celio-Panhysterectomy, George M. Edebohls, New York; Hysterectomy, Indications and Technique, J. M. Baldy, Philadelphia, Pa.; Notes pour l'Histoire des Fibromyomes Uterins, Nicholas San Juan, City of Mexico, Mexico; Vaginal Hysterectomy, E. E. Montgomery, Philadelphia, Pa.;

Total Extirpation of the Fibroid Uterus, Florian Krug, New York; A Plea for the Value of Early Diagnosis and Prompt Electrical Treatment of Fibroid Tumors of the Uterus, G. Betton Massey, Philadelphia, Pa.; The Results of Vaginal Hysterectomy, Andrew J. McCosh, New York; Cavernous Angioma of the Uterus Removed by Vaginal Hysterectomy (with Specimen), H. S. Boldt, New York.

AFTERNOON SESSION, SEPTEMBER 6TH.

The Treatment of Suppurative Disease of the Pelvic Organs, H. J. Boldt, New York; Drainage of Ovarian Cysts where the Adhesions are such that it is Impossible to Remove the Sac, A. Vanderveer, Albany, N. Y.; Post-operative Sequelæ of Pelvic and Abdominal Surgery, Joseph Price, Philadelphia, Pa.; After-treatment of Abdominal Section, L. S. McMurtry, Louisville, Ky.; The After-treatment of Coeliotomy Cases, with Special Reference to Shock and Sepsis, Eugene Boise, Grand Rapids, Mich.; Estudio Clinico sobre las Heridas Penetrantes del Abdomen i Pecho, Juan Manuel Escalana, Caracas, Venezuela; The Omentum and the Rôle it Plays in Operative Work upon the Abdomen, James F. W. Ross, Toronto, Can.; The Present Status of our Knowledge of the Pathology of Pelvic Inflammations, with Special Reference to the Treatment of Pelvic Abscess, R. B. Maury, Memphis, Tenn.

MORNING SESSION, SEPTEMBER 7TH.

An Inquiry into the Etiology of Mental Disturbances Following Operations upon the Pelvic Organs, George H. Rohé, Catonsville, Md.; What I Have Learned in the Surgery of the Gall-bladder, Joseph Eastman, Indianapolis, Ind.; Surgical Treatment of Peritonitis, M. B. Ward, Topeka, Kan.; A Study Based upon 100 Consecutive Cases of Removal of Diseased Uterine Appendages with two Deaths, R. Stansbury Sutton, Allegheny, Pa.; Report of 100 Operations Done for Serious Structural Disease of Abdominal and Pelvic Organs of Women, I. S. Stone, Washington, D. C.; Deductions from My First 110 Laparatomies for Appendicitis, with Report of Experimental Investigations, J. B. Murphy, Chicago, Ill.; Cura Radical de las Hernias, Luis C. Maglioni, Buenos Ayres; Last Resort in the Operative Treatment of Hernia, Robert T. Morris, New York.

AFTERNOON SESSION, SEPTEMBER 7TH.

Joint discussion by the Sections on Therapeutics, Surgery, and Gynecology on The Indications Governing the Employment of the Various Anesthetics. Co-referees for Section on Gynecology and Abdominal Surgery, G. M. Edebohls, New York, and E. E. Montgomery, Philadelphia.

MORNING SESSION, SEPTEMBER 8TH.

Apuntamientos para el Estudio Comparativo de la Pelvis Mexicana y Europea y Consecuencias Practicas á que dá Lugar la Especial Conformacion de la Primera, Manuel Gutierrez y Tomas Noriega, City of Mexico; The Dorsal Decubitus after Confinement and Miscarriages is the Most Frequent Cause of Retrodeviation with Fixation, A. Lapthorn Smith, Montreal, Can.; Ought Craniotomy to be Abolished? Wm. H. Myers, Fort Wayne, Ind.; Does the Catamenia Invariably Appear Earlier in Hot than in Cold Climates? J. H. O'Donnell, Winnipeg, Manitoba, Can.; When Operation is Refused, What Then? George R. Deane, Spartansburg, S. C.

Papers have also been promised by Edw. W. Jenks, Detroit, Mich.; W. E. B. Davis, Birmingham, Ala.; H. A. Kelly, Baltimore, Md., and others.

SECTION ON PATHOLOGY.

John Guiteras, M. D., Executive President, Philadelphia, Pa.; David Inglis, M. D., English-speaking Secretary, Detroit, Mich.; L. F. Criado, M. D., Spanish-speaking Secretary, Brooklyn.

Special attention of the profession is called to the practical demonstrations in pathology, photo-microscopy, and bacteriology.

One session devoted to a formal discussion on the subject of Cancer, to be opened by Dr. Wernicke, of Buenos Ayres, and, as co-referee, Prof. Allen J. Smith, of Galveston. Papers on this subject have been promised by Dr. Joshua M. Van Cott, Honorary President of the Section, and Dr. Joseph McFarland, of the Advisory Council.

Another session will be devoted to Yellow Fever, the discussion to be opened by Drs. Acosta and Grande, of Havana, Cuba, and, as co-referee, Dr. A. J. Amades, of Puerto Rico, Honorary President.

One day, two sessions, will be devoted to Practical Demonstrations, as follows: Dr. James E. Reeves, of Chattanooga, of the Advisory Council, Practical Demonstration of the Methods in Pathological Histology; Dr. Wm. M. Gray, of the Army Medical Museum, Practical Demonstration of the Methods in Photography Applied to Pathology; Dr. J. J. Kinyoun, P. A. Surg. U. S.

Marine Hospital Service, Practical Demonstration of Methods in Bacteriology.

Papers have been promised as follows: Notes on Three Years' Work in the Pathological Laboratory of the Charity Hospital of New Orleans, by Dr. Henry Dickson Burns, of New Orleans; Medical Geography of Puerto Rico, by Dr. A. J. Amades, of Puerto Rico; Theories of Inflammation, by Dr. Jose Torres Matos, of Havana; On Inflammation, by Dr. E. O. Shakespeare, of Philadelphia; On Cholera, by Dr. Herman M. Biggs, of New York; L'etat de Hyperexcitabilite du Nerf Phrenique, dans le Beribiri, by Dr. J. B. de Lacerda, of Rio de Janeiro; Paludismo, by Dr. A. J. Amades, of Puerto Rico; Bacteriological Observations on the Waters of the Harbor of Havana, by Drs. Acosta and Grande; Observations on Malaria, by Drs. Coronado and Madau; Operations of the Anti-rabic Laboratory in Havana, by Dr. Acosta; Abscess of the Liver, by Dr. James E. Reeves, of Chattanooga; On Influenza, by Dr. Ramon Guiteras, of New York; Observations on the Brains of Feeble-minded Children, by Dr. Henry W. Cattell; Pathology of Pelvic Inflammatory Trouble, by Dr. Joseph Price, Philadelphia.

Papers have been promised, without giving the subject, by Prof. Wm. H. Welch, of Baltimore; by Dr. W. J. Councilman, of Boston; and by Dr. G. F. H. Nuttall, of Baltimore, and Drs. Wm. Hughes and W. J. Carter, of Philadelphia.

---

American Dermatological Association.—Program of the seventeenth annual meeting, to be held at the Hotel Pfister, Milwaukee, Wis., September 5, 6, and 7, 1893:

*Officers for 1893.*—President, George Henry Fox, M. D., of New York; Vice-President, Henry W. Stelwagon, M. D., of Philadelphia; Secretary and Treasurer, George T. Jackson, M. D., of New York; Council: E. B. Bronson, M. D., G. T. Jackson, M. D., G. H. Fox, M. D., H. W. Stelwagon, M. D., J. C. White, M. D.

*First Day, Tuesday, September 5, 1893.*—Business meeting (with closed doors) at 9.30 A. M.; Report of the Council; Nomination of Officers for the ensuing year; Appointment of Auditing Committee; Proposals for Active and Honorary Membership; Miscellaneous Business.

Morning session, at 10.30 o'clock. President's address. Papers: Antiseptic Treatment of Skin Diseases, by Dr. C. W. Cutler; The Principles of Antisepsis in the Treatment of Eczema, by Dr. H.

G. Klotz; Cosmetics, by Dr. R. B. Morison. Adjournment at 1 P. M.

Evening session, at 8 o'clock. A Case of Tuberculosis of the Skin Simulating Lupus Erythematosus, by Dr. W. A. Hardaway; A Case of Rhinoscleroma, by Dr. G. T. Jackson; Atrophia Maculosa Cutis, with a Case, by Dr. W. T. Corlett.

*Second Day, Wednesday, September 6, 1893.*—Business meeting (with closed doors) at 9.30 A. M. Reports of Treasurer and Auditing Committee; Election of Officers; Report of the Council upon Candidates for Membership; Election of Active and Honorary Members; Selection of time and place of next meeting; Miscellaneous Business.

Morning session, at 10.30 o'clock. Report of Committee on Statistics; General Discussion on Pityriasis Rosea: (*a*) Its Etiology, (*b*) Its Relation to Ringworm, Seborrhea, Eczema, etc., (*c*) Its Treatment; Dermatitis Exfoliatava: (*a*) Its Clinical Forms, (*b*) Its Etiology, (*c*) Its Treatment; What do we Understand by Pemphigus? Adjournment at 1 P. M.

Evening session at 8 o'clock. A Contribution to the Pathology of Acne Varioliformis, by Dr. J. A. Fordyce; Angiokeratoma, by Dr. J. Zeisler; Subject to be announced, by Dr. M. B. Hartzell.

Dr. H. R. Crocker, of London, will read a paper on Lupus Erythematosus as an Imitator.

Retirement of old officers and induction of those newly elected. Adjournment.

It is earnestly requested that a duplicate copy of every paper read at the meeting be handed to the Secretary for publication in the transactions.

---

THE AMERICAN SOCIAL SCIENCE ASSOCIATION.—The Education Department of the American Social Science Association offers an unusually attractive program for its day at Saratoga, September 5, 1893. Mr. Hamilton W. Mabie, the well-known literateur and editor of the *Outlook*, will make the opening address. This will be followed by a paper on The Seamy Side of the Kindergarten, by Edward Fisher, of Berkshire, Mass. American Colleges and their Work, is Dr. G. Stanley Hall's subject. Dr. Louise Fiske Bryson will read a paper on The Education of Epileptics, and the Hon. Oscar Strauss will speak on Turkey and Civilization. In the Health Department, September 6th, Dr. Frederick Peterson will give an address on Recent Progress in Medicine and Surgery.

Pan-American Medical Congress.—The headquarters of the Section on Hygiene, Climatology, and Demography, with which the Section on Marine, Hygiene, and Quarantine has been consolidated, and of the Section on Military Medicine and Surgery, will be at the Hotel Richmond, corner of Seventeenth and H streets, N. W., Washington, the proprietor of which, Major F. W. Coleman, offers reduced rates to members and their families attending the Pan-American Medical Congress, September 5–8, 1893.

